# Editorial: Functions of liver and adipose tissue in metabolic disorder diseases of ruminants

**DOI:** 10.3389/fvets.2022.1009112

**Published:** 2022-09-14

**Authors:** Einar Vargas-Bello-Pérez, Xiliang Du, Guowen Liu, Jianguo Wang, Manuel Gonzalez-Ronquillo

**Affiliations:** ^1^Department of Animal Sciences, School of Agriculture, Policy and Development, University of Reading, Reading, United Kingdom; ^2^State Key Laboratory for Zoonotic Diseases, Key Laboratory of Zoonosis Research, Ministry of Education, College of Veterinary Medicine, Jilin University, Changchun, China; ^3^College of Veterinary Medicine, Northwest A&F University, Xianyang, China; ^4^Facultad de Medicina Veterinaria y Zootecnia, Departamento de Nutrición Animal, Instituto Literario 100 C.P. 50000, Universidad Autonoma del Estado de México, Toluca, Mexico

**Keywords:** adipose tissue, liver, ketosis, fatty acids, ruminants

In ruminants, most of the metabolic diseases (e.g., ketosis, fatty liver, ruminal acidosis) occur within the first month of lactation. For producers, these disorders represent enormous economic losses due to reductions in milk yield, increased risk of culling and mortality, and increased incidence and duration of common postpartum diseases (e.g., gastrointestinal disorders, displaced abomasum, and milk fever).

Liver is an essential organ, that plays an important role in preserving and regulating the metabolism of lipids, glucose, proteins in the body as well as energy metabolism. On the other hand, adipose tissue is a primary site for energy storage in the form of neutral triacylglycerol and controls systemic energy balance by regulating lipid mobilization and distribution in the body. Adipose tissue acts as an endocrine organ that produces numerous bioactive factors, such as TNF-α, IL-6, leptin, adiponectin and resistin, which communicate with other organs and affect metabolic homeostasis. In ruminants, a crosstalk between adipose tissue and liver is critical to keep animal's metabolic homeostasis. Thus, understanding liver and adipose tissue functions are pivotal in the context of preventing metabolic dysfunction of ruminants. Therefore, this Research Topic “Functions of liver and adipose tissue in metabolic disorder diseases of ruminants” aimed at compiling scientific reports focused on the alterations of liver and adipose tissue functions in the occurrence of metabolic disorder diseases of ruminants, the molecular mechanism of liver and adipose tissue dysfunction in ruminants, the crosstalk between liver and adipose tissue in the control of metabolic homeostasis of ruminants, and the potential strategy to detect and treat liver damage and adipose tissue dysfunction in the development and progress of ruminant metabolic dysfunction.

This Research Topic accepted seven articles covering the aforementioned aspects. Xue et al. assessed hepatic injury and described its metabolic mechanism in ruminants fed diets with different dietary energy levels. They used 25 Yunnan semi-fine wool sheep and fed them with five different dietary metabolic energy levels: low energy, medium–low energy, medium energy, medium–high energy, and high energy. They concluded that both high and low dietary energy levels caused hepatic injury and their data on liver tissue metabonomic analysis showed that hepatic injury might be caused by altered metabolism and lipid accumulation induced by lipid mobilization.

Transition dairy cows are often in negative energy balance leading to lipid mobilization and high serum β-hydroxybutyrate (BHBA) and non-esterified fatty acid (NEFAs) levels, which can later induce ketosis and fatty liver in dairy cows. Deng et al. determined the relationship between negative energy balance, insulin resistance and inflammation in dairy cows and assessed the role of non-esterified fatty acids in the nuclear factor kappa beta (NF-κB) inflammatory and insulin signaling pathways through Toll-like receptor 4 (TLR4). For that, primary calf hepatocytes were cultured and added different concentrations of NEFAs to assess the mRNA and protein levels of inflammatory and insulin signaling pathways. They reported that high-dose NEFAs (2.4 mM) can activate the TLR4/NF-κB inflammatory signaling pathway and reduce the sensitivity of the insulin pathway through the TLR4/PI3K/AKT metabolic axis.

It has been suggested that during early lactation and the transition period, higher plasma growth hormone (GH) levels in subclinical ketosis (SCK) might involve the initiation of body adipose tissues mobilization, resulting in metabolic disorders in ruminants particularly hyperketonemia. Mohsin et al. characterized plasma levels of GH, β-hydroxybutyrate acid (BHBA) and non-esterified fatty acid (NEFA) and glucose in ketotic cows and healthy control (CON) cows; to measure the liver function test indices in ketotic and healthy CON cows. Their study concluded that during postpartum, higher plasma GH levels in SCK cows might involve the initiation of body adipose tissue mobilization, resulting in hyperketonemia. In the same line, Yu et al. determined whether the enhanced transcription factor EB (TFEB) transcriptional activity contributes to lipolysis of adipose tissue in SCK cows and explored the possibility of establishing a therapeutic strategy by using TFEB as a target to control lipolysis. Interestingly, their findings indicated that enhanced transcriptional activity of TFEB may contribute to lipolysis of adipose tissue in dairy cows with SCK. The regulation of TFEB activity may be an effective therapeutic strategy for controlling overt lipolysis in ketotic cows.

Essential oils extracted from plants contain secondary metabolites that exhibit anti-inflammatory activity and tea tree oil (TTO) has been shown to play an important role in lipid metabolism, as it ameliorates inflammatory responses. Thus, Yang et al. performed a study to determine if TTO alleviates palmitic acid (PA)-induced lipid accumulation in bovine hepatocytes. Hepatocytes isolated from mid-lactating Holstein cows were used for this purpose. In summary, they showed that TTO exerts anti-inflammatory effects and acts to promote lipid homeostasis in bovine hepatocytes. These effects were associated with inhibition of NF-κB signaling and SREBP1c expression. Their data suggests that TTO treatment may be a promising therapeutic approach to imbalanced lipid homeostasis, inflammation, and endoplasmic reticulum stress in dairy cows shortly before and after calving.

Reichelt et al. investigated the suitability of the fetal bovine hepatocyte-derived cell line BFH12 as a model for hepatosteatosis which is a common metabolic disorder of dairy cows, especially during early lactation. They found that BFH12 may be a useful *in vitro* model to study bovine hepatosteatosis and its underlying molecular mechanisms.

Lastly, in a brief research report, Stiensmeier and Schmicke elucidated if the active form of thyroid hormones (T3) may have an additive and direct effect on hepatic GHR mRNA expression *in vitro* in bovine primary hepatocytes. With that study, they confirmed that T3 has a stimulatory effect on GHR1A mRNA expression at least at *in vitro* levels.

In summary, results from the above-mentioned studies have improved our understanding on the functions of liver and adipose tissue in metabolic disorder diseases of ruminants. Despite all existing literature related to this topic. Many efforts are still needed to elucidate way for preventing metabolic problems during the first weeks after parturition in dairy ruminants, especially in cattle.

## Author contributions

All authors listed have made a substantial, direct, and intellectual contribution to the work and approved it for publication.

## Conflict of interest

The authors declare that the research was conducted in the absence of any commercial or financial relationships that could be construed as a potential conflict of interest.

## Publisher's note

All claims expressed in this article are solely those of the authors and do not necessarily represent those of their affiliated organizations, or those of the publisher, the editors and the reviewers. Any product that may be evaluated in this article, or claim that may be made by its manufacturer, is not guaranteed or endorsed by the publisher.

